# Modulation of Inflammation by Alcohol Exposure

**DOI:** 10.1155/2014/283756

**Published:** 2014-02-05

**Authors:** Mark Lehnert, Elizabeth J. Kovacs, Patricia E. Molina, Borna Relja

**Affiliations:** ^1^Department of Trauma, Hand and Reconstructive Surgery, J.W. Goethe University, 60323 Frankfurt, Germany; ^2^Alcohol Research Program, Department of Surgery and Department of Microbiology and Immunology, Burn & Shock Trauma Institute, Loyola University Chicago, Maywood, IL 60153, USA; ^3^Department of Physiology, Louisiana State University Health Sciences Center, New Orleans, LA 70112, USA

The morbidity and mortality resulting from alcohol-related diseases globally impose a substantive cost to society [1, 2]. Alcohol consumption is a major risk factor for all types of injuries [3] and excessive alcohol consumption is the third leading cause of preventable death worldwide. Besides its multiorgan system effects, ethanol exposure is known to cause changes in the physiological response following inflammatory stimuli leading to increased morbidity and mortality. Multiple studies have demonstrated that alcohol significantly affects the immune system and that modulation of inflammatory reactions seems not only to depend upon the pattern of exposure (acute ethanol intoxication—binge like ethanol consumption—chronic ethanol abuse) but also differs depending on the underlying insult that causes the inflammatory reactions (i.e., hemorrhagic shock-burns) [4]. In the present special issue, 5 original research reports as well as a clinical study and a review report elucidate the influence of alcohol on the pathophysiology of inflammation. This featured topic issue contains reports that highlight the impact of alcohol on responses to traumatic injury. In addition, it features reports describing the inflammatory responses associated with alcoholic liver disease and the impact of alcohol on lung epithelial cell function. Together these reports add to our body of knowledge of the significant detrimental impact of alcohol on systemic responses to injury and on its contribution to disease progression. The study-called “*Heavy ethanol intoxication increases proinflammatory cytokines and aggravates hemorrhagic shock-induced organ damage in rats*” by T. M. Hu and colleagues demonstrates that the high levels of proinflammatory mediators generated after the combined insult of acute ethanol administration and hemorrhagic shock cause both immunosuppression and resulted in excessive tissue damage relative to either insult alone. These studies examined liver, kidney, and pulmonary, responses.

In a study by M. M. Chen, entitled “*Intoxication by intraperitoneal injection or oral gavage equally potentiate postburn organ damage and inflammation*” reveals that the route of administration of alcohol (oral gavage versus intraperitoneal injection) similarly effects postburn responses. M. M. Chen et al. found that intoxication potentiates postburn damage in the ileum, liver, and lungs of mice to an equivalent extent when either ethanol administration route is used. Moreover, they showed comparable systemic changes including the hematologic response and serum levels of proinflammatory mediators, including interleukin-6 (IL-6).

In a review entitled “*The effect of inflammatory cytokines in alcoholic liver disease*,” H. Kawaratani and colleagues compared the roles of pro- and anti-inflammatory mediators in the liver in a rat model of alcoholic liver disease. Their model suggests that cytokines, chemokines, oxidative stress, and microbial flora play a role in the development and progression of alcoholic liver disease. The review describes the link between the derived endotoxins and the activation of Kupffer cells through the LPS/Toll-like receptor (TLR) 4 pathways.

“*The study called Association of serum adiponectin, leptin and resistin concentrations with the severity of liver dysfunction and the disease complications in alcoholic liver disease*” by B. Kasztelan-Szczerbinska and coworkers investigate serum levels of adiponectin, leptin, and resistin in patients with chronic alcohol abuse and different grades of liver dysfunction, as well as alcoholic liver disease (ALD) complications in inpatients. Their data revealed both changes in some of these mediators and in difference in sex, which shows that the leptin concentrations may play a role in the observed sex difference in the development of ALD.

“*The study called Alcohol-induced liver injury is modulated by Nlrp3 and Nlrc4 inflammasomes in mice*” by D. A. DeSantis and colleagues reported on the role of members of the Nod-like receptor (NLR) family in the development of alcoholic liver disease. The authors utilized gene targeted deletions for Nlrp3 (Nlrp3^−/−^) and Nlrc4 (Nlrc4^−/−^) in chronic alcohol consumption model and their data suggest that the Nlrp3 inflammasome is protective during alcohol induced liver injury.

M. Lehnert and his group describe how chronic ethanol modulates inflammatory mediators, activation of nuclear factor-*κ*B in murine Kupffer cells, and circulating leukocytes. They investigated the effect of chronic ethanol feeding on *in vivo* activation of NF-*κ*B in circulating leukocytes and whole liver of NF-*κ*B-EGFP reporter gene mice. The work focuses on the production of proinflammatory mediators and activation of NF-*κ*B in Kupffer cells after chronic ethanol feeding followed by *in vitro* stimulation with lipopolysaccharide (LPS).

Shifting to the lung, Todd Wyatt and his group report on the effects of “exhalation of volatized ethanol from the bronchial circulation on bronchial epithelial cells.” Their data reveal that alcohol exposure leads to an early elevation in the cilia beat frequency of bronchial epithelial cells followed by a chronic desensitization of cilia stimulatory responses. This effect is controlled, in part, by nitric oxide mediated protein kinase A activation.

Taken together, this special issue highlights up to date research on the pathophysiology of alcohol associated inflammation. Every paper opens the door to further hypothesis-driven investigations emphasizing the need for ongoing preclinical and clinical studies.

*Mark Lehnert* *Mark Lehnert* *Elizabeth J. Kovacs* *Elizabeth J. Kovacs* *Patricia E. Molina* *Patricia E. Molina* *Borna Relja* *Borna Relja*
